# Dietary DHA/EPA Ratio Changes Fatty Acid Composition and Attenuates Diet-Induced Accumulation of Lipid in the Liver of ApoE^−/−^ Mice

**DOI:** 10.1155/2018/6256802

**Published:** 2018-11-14

**Authors:** Liang Liu, Qinling Hu, Huihui Wu, Xiujing Wang, Chao Gao, Guoxun Chen, Ping Yao, Zhiyong Gong

**Affiliations:** ^1^Key Laboratory for Deep Processing of Major Grain and Oil, Ministry of Education, Wuhan 430023, China; ^2^College of Food Science and Engineering, Wuhan Polytechnic University, Wuhan 430023, China; ^3^Hubei Key Laboratory for Processing and Transformation of Agricultural Products (Wuhan Polytechnic University), Wuhan 430023, China; ^4^National Institute for Nutrition and Health, Chinese Center for Disease Control and Prevention, Beijing 100050, China; ^5^Department of Nutrition, University of Tennessee at Knoxville, Knoxville 37996, USA; ^6^Department of Nutrition and Food Hygiene, School of Public Health, Tongji Medical College, Huazhong University of Science and Technology, Wuhan 430030, China

## Abstract

Diets containing various docosahexaenoic acid (DHA)/eicosapentaenoic acid (EPA) ratios protect against liver damage in mice fed with a high-fat diet (HFD). However, it is unclear whether these beneficial roles of DHA and EPA are associated with alterations of fatty acid (FA) composition in the liver. This study evaluated the positive impacts of n-6/n-3 polyunsaturated fatty acids (PUFAs) containing different DHA/EPA ratios on HFD-induced liver disease and alterations of the hepatic FA composition. ApoE^−/−^ mice were fed with HFDs with various ratios of DHA/EPA (2 : 1, 1 : 1, and 1 : 2) and an n-6/n-3 ratio of 4 : 1 for 12 weeks. After treatment, the serum and hepatic FA compositions, serum biochemical parameters, liver injury, and hepatic lipid metabolism-related gene expression were determined. Our results demonstrated that dietary DHA/EPA changed serum and hepatic FA composition by increasing contents of n-6 and n-3 PUFAs and decreasing amounts of monounsaturated fatty acids (MUFAs) and the n-6/n-3 ratio. Among the three DHA/EPA groups, the DHA/EPA 2 : 1 group tended to raise n-3 PUFAs concentration and lower the n-6/n-3 ratio in the liver, whereas DHA/EPA 1 : 2 tended to raise n-6 PUFAs concentration and improve the n-6/n-3 ratio. DHA/EPA supplementation reduced the hepatic impairment of lipid homeostasis, oxidative stress, and the inflammatory responses in HFD-fed mice. The DHA/EPA 2 : 1 group had lower serum levels of total cholesterol, triglycerides, and low-density lipoprotein cholesterol and higher levels of adiponectin than HFD group. The DHA/EPA 1 : 2 group had elevated serum levels of aspartate aminotransferase, alanine aminotransferase, and alkaline phosphatase, without significant change the expression of genes for inflammation or hepatic lipid metabolism among the three DHA/EPA groups. The results suggest that DHA/EPA-enriched diet with an n-6/n-3 ratio of 4 : 1 may reverse HFD-induced nonalcoholic fatty liver disease to some extent by increasing n-6 and n-3 PUFAs and decreasing the amount of MUFAs and the n-6/n-3 ratio.

## 1. Introduction

Nonalcoholic fatty liver disease (NAFLD), the most common chronic liver disease worldwide, is one of the major causes of the fatty liver, occurring when fat is deposited in the liver in the absence of excessive alcohol intake [1, 2]. Currently, its prevalence in Asia is estimated to be 25%, similar to the incidence in many western countries (20–30%), and is even as high as 40% in westernized Asian populations [3]. The development of NAFLD is directly associated with enhancement in prooxidant status [4], proinflammatory status [5], and lipid content [4, 6] of the liver in mice fed with a high-fat diet (HFD) [7].

Lifestyle modification, including dietary changes, weight loss, and physical activity, is the initial treatment option for patients with NAFLD [8]. On the other hand, dietary modification may benefit the treatment of NAFLD without significant weight loss [9]. Accumulating clinical evidence has revealed that low levels of n-3 polyunsaturated fatty acids (n-3 PUFAs), including *α*-linolenic acid (ALA), in serum and liver tissue samples are common characteristics of patients with alcoholic disease and NAFLD [10, 11], which may be attributed to impaired bioavailability of liver n-6 and n-3 PUFAs [11–13]. Jump et al. [14] provided an in-depth rationale for the use of dietary n-3 PUFA supplements as a treatment option for NAFLD. Experimental and clinical data on n-3 PUFAs have also demonstrated that dietary supplementation with eicosapentaenoic acid (EPA, C20:5) and docosahexaenoic acid (DHA, C22:6) prevents or alleviates NAFLD [15]. Additionally, a recent transcriptomic study showed that fish oil protected against HFD- and high-cholesterol diet-induced NALFD by improving lipid metabolism and ameliorating hepatic inflammation in Sprague-Dawley rats [16]. We also reported that diet rich in DHA and/or EPA improved lipid metabolism and had anti-inflammatory effects in HFD-induced NALFD in C57BL/6J mice [17]. Thus, daily intake of DHA and EPA for healthy adults as well as those with coronary artery diseases and hypertriglyceridemia is strongly recommended by authority organizations. However, the precise requirement for marine n-3 PUFAs is not known [9].

Recently, the effects of different dietary n-6/n-3 ratio on health and disease have drawn close attention. A higher intake of n-6 FA and higher dietary n-6/n-3 FA ratio were reported in NAFLD subjects [18]. On the other hand, additional evidence also highlighted the role of ratios of DHA and EPA in the prevention and treatment of chronic disease in rat models [19–22], indicating the importance of both n-6/n-3 ratios and DHA/EPA ratios. It has been known that the intake of dietary fat alters the FA composition of plasma and various organs, including the liver [12, 18]. Lipidomics analysis has also revealed the role of different EPA/DHA ratios in the modulation of inflammation and oxidative markers in genetically obese hypertensive rats through the downregulation of the production of proinflammatory n-6 eicosanoids [23]. We previously showed that an oral administration of n-6/n-3 PUFAs with varying DHA/EPA ratios for 12 weeks ameliorated atherosclerosis lesions [24] and liver damage [17] in mice fed with an HFD. Data from aforementioned studies suggested the positive effects of supplementation with varying DHA/EPA ratios on the metabolic parameters of HFD-fed animals. However, there have been few studies on the protective role of n-6/n-3 PUFA supplementation with varying DHA/EPA ratios against HFD-induced liver damage and its correlation with hepatic FA composition.

Therefore, the focus of this study was to evaluate the positive effects of n-6/n-3 PUFA supplementation with varying DHA/EPA ratios on liver disease induced by an HFD as well as the associated alterations of FA composition of the liver.

## 2. Materials and Methods

### 2.1. Animals and Diets

Male apolipoprotein E knockout (*ApoE*^−/−^) mice at weaning (C57/BL6 background, 6 weeks old, 20 ± 2 g) were obtained from Vital River Laboratories (Beijing, China). All of the mice were housed in a humidity and temperature controlled room (relative humidity, 65–75%; temperature, 20–24°C) with a 12 h : 12 h light/dark cycle and were given *ad libitum* access to their specific diets and water. After a 1-week acclimation, the mice were randomly divided into the following five groups: (1) normal diet (ND) group (control group received an ND of basic feed 86%, casein 4%, and yolk powder 10%), (2) HFD group received HFD I (basic feed 70%, 15% lard, 1% cholesterol, casein 4%, and yolk powder 10%), and (3–5) DHA/EPA groups (2 : 1, 1 : 1, and 1 : 2) received HFD II (basic feed 75%, 10% lard, 1% cholesterol, casein 4%, and yolk powder 10%) plus mixed oil. The mixed oil (including sunflower seed, perilla, fish, and algal oils) was formulated by the previous method [24] for partial replacement of 5% lard, with adjustment of the n-6/n-3 ratio to 4 : 1 and with variation in the DHA/EPA ratios (2 : 1, 1 : 1, and 1 : 2). The diets were prepared according to the previous method [17, 24]. The FA profiles of oils, basic feed, and HFDs were quantified by gas chromatography [24]. The FA compositions of oils, basic feed, control diet, and HFDs are shown in Table 1. The lipids were administered orally (1 g/kg body weight (BW)) for 12 weeks. The ND and HFD groups were given the same dose of physiological saline via intragastric administration. Their BWs were recorded once a week. The *Guide for the Care and Use of Laboratory Animals* by the National Institutes of Health (Bethesda, MD, USA) was followed during the experiments [25]. The animal protocol was approved by the Tongji Medical College Council on Animal Care Committee (Wuhan, China). At the end of the experiments, mice after 12 h of fasting were anesthetized with isoflurane before blood and tissue sample collections. Serum was collected from blood after agglutination and centrifugation at 4000 ×g at 4°C for 10 min and then stored at −80°C. Fresh tissue samples were fixed for histopathology determinations or were quick-frozen in liquid nitrogen for quantitative PCR (qPCR) and western blot analyses.

### 2.2. Lipid Extraction and FA Analysis

Total lipid from serum or liver tissue homogenates was extracted with ice-cold chloroform/methanol (2 : 1 *v*/*v*) with 0.01% butylated hydroxytoluene. After centrifugation, the phase interface was washed with chloroform/methanol/water (3 : 48 : 47 *v*/*v*/*v*). Methyl esterification of the lipids was conducted according to the previous report [26]. Fatty acid methyl esters (FAMEs) were quantified using the Agilent Technologies 6890 Gas Chromatograph (Agilent Technologies Inc., Savage, MD, USA) with a flame ionization detector. Separation of the FAMEs was performed on the HP-INNOWax capillary column (30 × 0.32, 0.25 *μ*m; Agilent) using helium as carrier gas at a constant flow of 1.5 mL/min. The samples were injected at a starting oven temperature of 50°C; the injector and detector temperatures were 250°C. The oven temperature was programmed as follows: 50°C, 1 min, 15°C/min to 175°C, 5 min, and 1°C/min to 250°C. The FAMEs were identified by comparing with authentic standards (Nu-Chek-Prep) and were calculated as the percent area of total FAs.

### 2.3. Histopathological Analysis

Fresh liver slices were processed by hematoxylin and eosin (H&E) staining. Briefly, liver tissues were cut into slices and fixed, and then, the samples were dehydrated and embedded with paraffin. Paraffin-embedded tissue sections (5 *μ*m) were stained with H&E and observed under the Olympus BX50 light microscope (Olympus, Tokyo, Japan).

### 2.4. Measurements of Serum Parameters and Fat Liver Content

Serum total cholesterol (TC, mM), triglyceride (TG, mM), low-density lipoprotein cholesterol (LDL-C, mM), high-density lipoprotein cholesterol (HDL-C, mM) levels, and hepatic TC (mM/g protein) and TG (mM/g protein) were determined by spectrophotometric methods using the respective kits (Biosino Biotechnology Co. Ltd., Beijing, China) according to the manufacturer's instructions. Serum aspartate transaminase (AST, U/L), alanine transaminase (ALT, U/L), and alkaline phosphatase activities (AKP, U/L) were measured using specific diagnostic kits (Nanjing Jiancheng Corporation, Nanjing, China). Enzyme-linked immunoassay (ELISA) kits were used to assess the serum levels of tumor necrosis factor alpha (TNF-*α*, pg/mL), interleukin-1*β* (IL-1*β*, pg/mL), and adiponectin (Cloud-Clone Corp., Wuhan, China).

### 2.5. Analysis of Hepatic Malondialdehyde, Superoxide Dismutase, and Glutathione

Hepatic malondialdehyde (MDA, *μ*M/g protein), glutathione (GSH, *μ*M/g protein), and superoxide dismutase (SOD, U/mg protein) were determined using the respective kits (Nanjing Jiancheng Corporation, Nanjing, China).

### 2.6. qPCR Analysis

Total RNA of mouse liver samples was extracted using the TRIzol reagent (Ambion®, Life Technologies, Austin, TX, USA) according to the manufacturer's instructions. Messenger RNA (mRNA) expression levels of the target genes were quantified using the SYBR Green-based Kit (Takara Bio Inc., Dalian, China) with specific primers and a real-time PCR machine for qPCR (IQ5; Bio-Rad, Hercules, CA, USA). The mRNA level of *β*-actin was used as the invariable control for quantification, and the results were calculated by the comparative 2^−ΔΔCt^ method. The sequences of the forward and reverse primers used for the detection of the target genes are listed in Table 2.

### 2.7. Western Blot Analysis

The liver tissues were homogenized in radioimmunoprecipitation assay lysis buffer (1% Triton X-100, 1% deoxycholate, and 0.1% sodium dodecyl sulfate (SDS)), and protein concentration was measured. Equal amounts of protein extracts were mixed (3 : 1, *v*/*v*) and processed in loading buffer for electrophoresis in 10% acrylamide SDS gels and subsequently electroblotted to a nitrocellulose transfer membrane (Merck Millipore, Burlington, MA, USA) using a Trans-Blot SD semidry electrophoretic transfer cell (Bio-Rad). Target proteins were probed with specific primary antibodies, and then, the bound primary antibodies were recognized with species-specific secondary antibodies. The chemiluminescence intensity of the specific proteins on the membrane was subsequently detected using the SuperSignal West Pico Chemiluminescent Substrate (Thermo Fisher Scientific, Waltham, MA, USA) and a western blotting detection system (Bio-Rad). The optical densities (OD) of the bands were quantified using the Gel-Pro 3.0 software (Biometra, Goettingen, Germany). The density of the specific protein band was corrected to eliminate background noise and normalized to that of GAPDH (Boster Biological Technology Ltd., Wuhan, China) as OD/mm^2^.

### 2.8. Statistical Analysis

Statistical analysis was performed with the GraphPad Prism 4.0.3 software (GraphPad Prism Software Inc., San Diego, USA). Data were presented as mean ± standard error of the mean (SEM). One-way analysis of variance was performed with Fisher's least significant difference multiple comparison post hoc test. A *P* < 0.05 was considered statistically significant.

## 3. Results

### 3.1. Dietary DHA/EPA Reduces HFD-Induced Liver Injury

**Figure 1:**
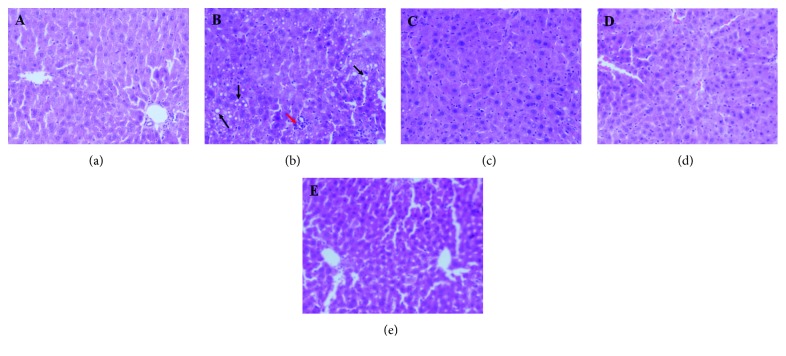
Effects of the supplementation of various DHA/EPA ratios on hepatic lipid metabolism. H&E staining of liver sections in each group, followed by observation under a light microscope (magnification 200x). Notice the fatty vesicles (black arrow) and lymphocyte infiltration (red arrow). (a) Normal diet (ND) group, (b) high-fat diet (HFD) group, (c) DHA/EPA 2 : 1 (DHA/EPA = 2 : 1) group, (d) DHA/EPA 1 : 1 (DHA/EPA = 1 : 1) group, and (e) DHA/EPA 1 : 2 (DHA/EPA = 1 : 2) group.

Treatment with DHA/EPA did not change the BWs and liver weights in the study. The mice in the five dietary groups showed similar initial BWs, final BWs, and liver/BW ratio (Table 3). The hepatic histological changes were observed by light microscopy of tissue sections with stained H&E (Figure 1). The main change that occurred in the liver from the HFD group was macrovesicular steatosis, as determined by the observation of lipid vesicles in the cytosolic compartment, along with neutrophil and lymphocyte infiltration. However, DHA/EPA-supplemented mice were much fewer and smaller hepatic fatty vesicles than the HFD group mice did.

As illustrated in Table 4, compared with ND-fed mice, serum levels of AST, ALT, and AKP levels were higher (*P* < 0.05) in HFD-fed mice. However, various ratios of DHA/EPA supplementation significantly alleviated HFD-induced liver injury by reducing serum levels of AST (ranging from 71.6% to 86.9%), ALT (ranging from 66.6% to 80.7%), and AKP (ranging from 22.4% to 53.6%). No significant change was observed in the activities of serum aminotransferases among the DHA/EPA groups; AST, ALT, and AKP levels were highest in the DHA/EPA 1 : 2 group.

Hepatic MDA was significantly boosted in HFD-fed mice compared to that in the ND-fed mice (Table 4). The MDA production was markedly decreased by DHA/EPA supplementation. However, the inhibitory effects of different DHA/EPA ratios on MDA production were not significantly different (Table 4). In contrast to that in the HFD group, serum levels of GSH (increased more than 2-fold) and SOD (increased by 18.5%) were notably elevated in DHA/EPA-treated mice (Table 4). However, no significant differences of MDA, SOD, and GSH among the three DHA/EPA ratios were observed.

### 3.2. Dietary DHA/EPA Changes FA Composition of the Serum and Liver

FA compositions of the serum and liver samples in mice after the 12-week feeding of the HFD are shown in Tables 5 and 6, respectively. When the FA compositions of total liver lipids were compared, a significant decrease (*P* < 0.05) of total saturated fatty acids (SFAs) was observed in the HFD group compared with that in the ND group. This trend occurred in the abundance of total PUFAs (26.7% difference) (*P* < 0.001), including total n-6 and n-3 PUFAs (16.6% and 54.7% difference, respectively) with an 84.9% increase in the ratio of n-6/n-3. Also, the content of total MUFAs was significantly increased (*P* < 0.01) due to significant increases in 16 : 1 (palmitoleic acid) and C18:1 (oleic acid; 130% difference).

Among the varying ratios of DHA/EPA groups, we found an increase in SFAs (DHA/EPA 2 : 1 group, 19.6%; DHA/EPA 1 : 1 group, 14.5%), PUFAs n-6 series (DHA/EPA 2 : 1 group, 11.1%; DHA/EPA 1 : 1 group, 9.1%; and DHA/EPA 1 : 2 group, 17.9%), and PUFA n-3 series (DHA/EPA 2 : 1 group, 166.4%; DHA/EPA 1 : 1 group, 151.7%; and DHA/EPA 1 : 2 group, 126.3%) in the liver compared to the HFD group. Also, the amount of MUFAs (DHA/EPA 2 : 1 group, 49.7%; DHA/EPA 1 : 1 group, 41.8%; and DHA/EPA 1 : 2 group, 35.3%) and the ratio of n-6/n-3 (DHA/EPA 2 : 1 group, 58.3%; DHA/EPA 1 : 1 group, 55.9%; and DHA/EPA 1 : 2 group, 48.1%) showed a marked decrease after DHA/EPA supplementation. Among the three DHA/EPA groups, DHA/EPA 1 : 2 group had the lowest C18:0 and C20:1 concentration and the highest C18:2 and n-6 PUFA concentration. The DHA/EPA 2 : 1 group showed a tendency to raise n-3 PUFA concentration and lower SFAs, C20:5 and C22:0 concentrations, and n-6/n-3 ratio.

Concerning serum FA composition, the same trend was observed for the amount of MUFAs, PUFAs n-6 series, PUFAs n-3 series, and the ratio of n-6/n-3 in the three DHA/EPA groups compared with the HFD group. However, no significant difference among the three DHA/EPA ratios was found for the amount of SFAs, MUFAs, PUFAs n-6 series, and the ratio of n-6/n-3.

### 3.3. Dietary DHA/EPA Ameliorates HFD-Induced Hepatic Inflammation

**Figure 2:**
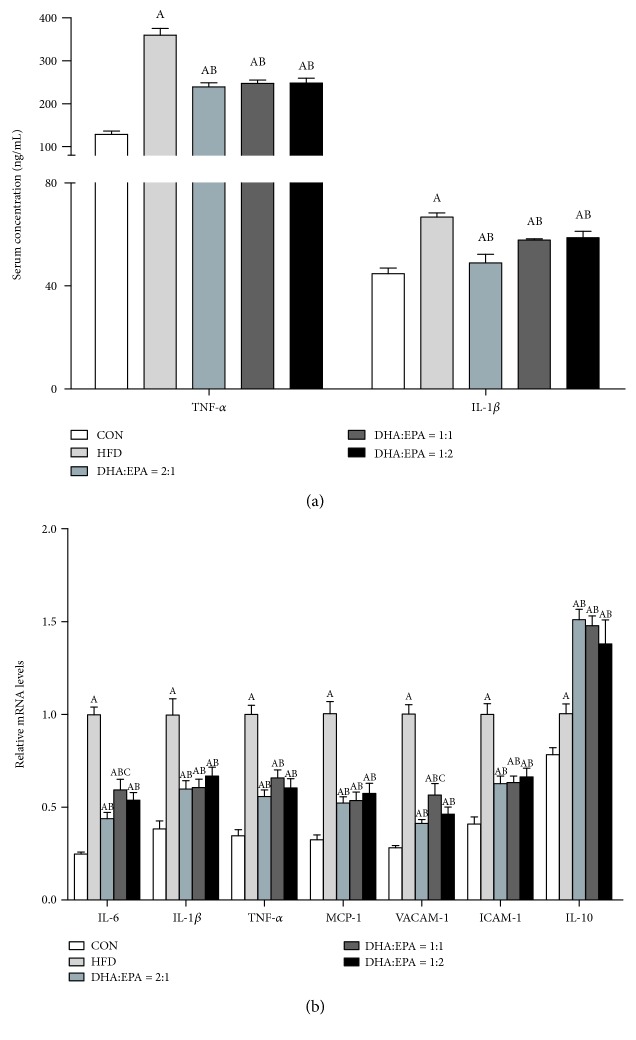
Effects of the supplementation of various DHA/EPA ratios on serum and hepatic inflammatory cytokine expression. (a) Serum inflammatory cytokines (*n* = 8). (b) Hepatic inflammatory cytokine expression (*n* = 6). The mRNA expression of *β*-actin was quantified as the endogenous control. (A) *P* < 0.05 versus the ND group; (B) *P* < 0.05 versus the HFD group; (C) *P* < 0.05 versus the DHA/EPA = 2 : 1 group; (D) *P* < 0.05 versus the DHA/EPA = 1 : 1 group.

The serum concentrations of both IL-1*β* and TNF-*α* were significantly lower in the three DHA/EPA-treated groups than those in the HFD group (Figure 2). In DHA/EPA-treated mice, the TNF-*α* level decreased by more than 30%. A similar trend was observed for serum levels of IL-1*β*. Consistent with findings for serum levels of proinflammatory cytokines, the data of qPCR analysis demonstrated significantly reduced hepatic expression levels of IL-6, IL-1*β*, TNF-*α*, monocyte chemoattractant protein-1 (MCP-1), vascular cell adhesion molecule-1 (VCAM-1), and intercellular adhesion molecule-1 (ICAM-1) in DHA/EPA-treated mice compared to those in the HFD-treated mice (Figure 2). The mRNA expression levels of the anti-inflammatory cytokine IL-10 were increased by 51.0%, 47.8%, and 38.0% in mice treated with DHA/EPA ratios of 1 : 2, 1 : 1, and 2 : 1, respectively.

### 3.4. Dietary DHA/EPA Improves HFD-Induced Lipid Dyshomeostasis in Liver Tissue

DHA/EPA treatment for 12 weeks resulted in a significant reduction in serum levels of TC (reduced by 46.9–72%), TG (reduced by 45.4–75.6%), LDL-C (reduced by 38.3–63.7%), and ox-LDL (reduced by 36.2–38.3%) compared to the HFD group (Table 4). Although the reduction effects of DHA/EPA on the hepatic lipid level have no significant difference among the three DHA/EPA groups, daily DHA/EPA treatment alleviated hepatic fatty accumulation. Moreover, the three groups treated with DHA/EPA had higher serum levels of HDL-C (increased by 61.5–169.2%) and adiponectin (increased by 27.4–141%) than the HFD group did. In particular, DHA/EPA 1 : 2 group had the lowest serum TC, TG, and LDL levels and the highest adiponectin level among the three DHA/EPA groups.

**Figure 3:**
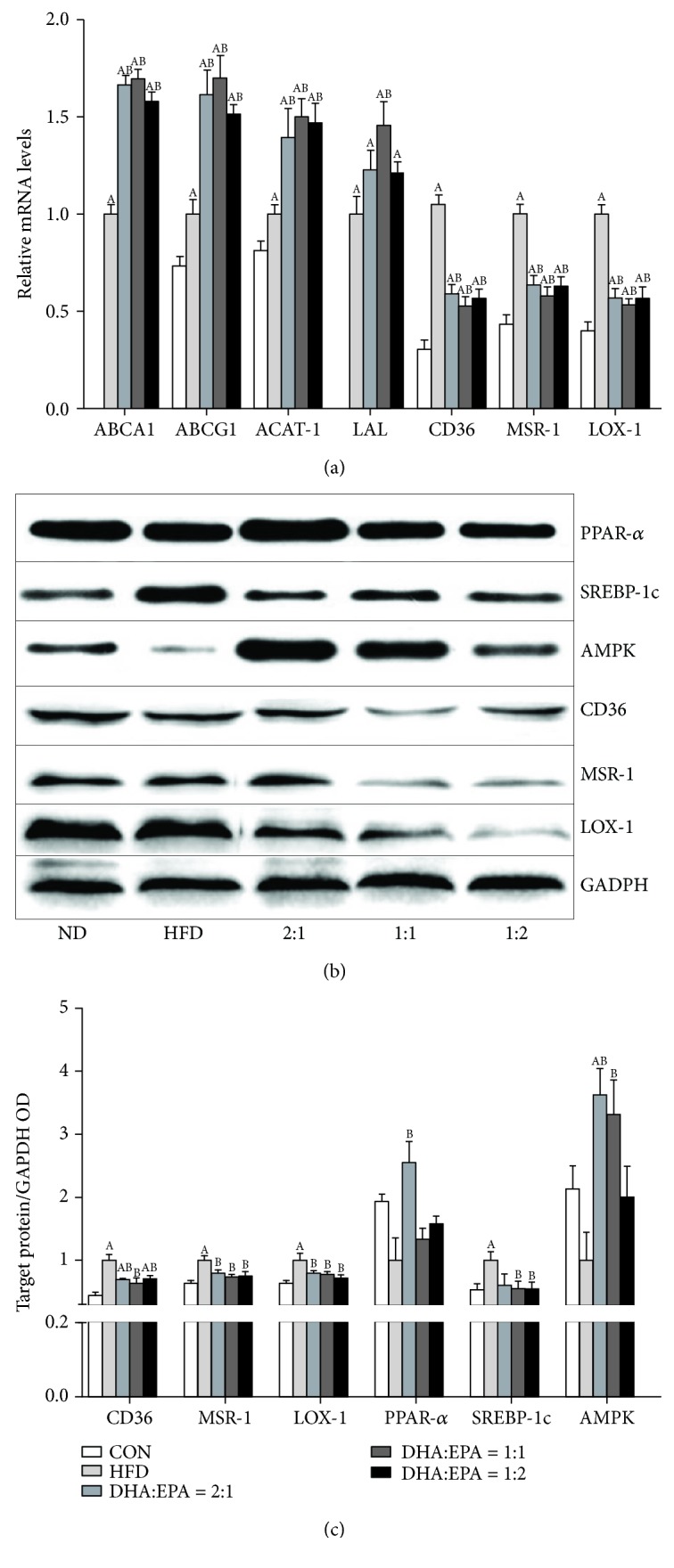
Effects of the supplementation of various DHA/EPA ratios on hepatic lipid metabolism. (a) The mRNA expression of ABCA1, ABCG1, ACAT-1, LAL, CD36, MSR-1, and LOX-1 in liver tissues, as measured by qPCR (*n* = 6). (b, c) Protein expression of PPAR*α*, SREBP-1c, AMPK, CD36, MSR-1, and LOX-1 in liver tissues, as measured by western blotting (*n* = 3–4). (A) *P* < 0.05 versus the ND group; (B) *P* < 0.05 versus the HFD group; (C) *P* < 0.05 versus the DHA/EPA = 2 : 1 group; (D) *P* < 0.05 versus the DHA/EPA = 1 : 1 group.

As illustrated in Figure 3, 66.5%, 69.7%, and 58.0% increases in the mRNA expression of ATP-binding cassette transporter A1 (ABCA1) were, respectively, observed in the DHA/EPA 1 : 2, DHA/EPA 1 : 1, and DHA/EPA 2 : 1 groups, compared with that in the HFD-treated mice (Figure 3). No significant difference of the ABCA1 expression level was found among the DHA/EPA groups. Compared to that in the HFD group, the same trend was observed in ATP-binding cassette transporter G1 (ABCG1) and acyl-coenzyme A:cholesterol acyltransferase (ACAT-1) in the DHP/EPA groups, although only the DHA/EPA 1 : 1 group showed a significant increase in lysosomal acid lipase (LAL) (*P* < 0.05). In liver tissue, cluster of differentiation 36 (CD36), macrophage scavenger receptor 1 (MSR-1), and lectin-like oxidized low-density lipoprotein receptor 1 (LOX-1) expression levels were significantly downregulated at both the mRNA and protein levels in DHA/EPA-treated mice compared to that in the HFD-treated mice. Additionally, the feeding of the HFD significantly downregulated the protein levels of proliferator-activated receptor alpha (PPAR*α*) and adenosine monophosphate-activated protein kinase (AMPK) and upregulated the protein levels of sterol regulatory element-binding protein 1c (SREBP-1c), compared with that of the ND, which were partially reversed with the supplementation of dietary DHA/EPA (Figure 3).

## 4. Discussion

Dietary n-3 PUFAs can reduce hepatic inflammation, fibrosis, and steatosis, decrease plasma TG concentrations, and regulate hepatic fatty acid and TG metabolism in NAFLD. We previously created a mouse model in which NAFLD, lipid disorder, oxidative stress, and inflammation were induced by an HFD in C57BL/6J mice [17]. Our findings showed that the consumption of diets with various ratios of DHA/EPA (2 : 1, 1 : 1, and 1 : 2) ameliorated liver steatosis in mice. This is probably due to the repletion of hepatic total n-3 PUFA content and decrease of the n-6/n-3 ratio, concomitant with a reduction of oxidative stress, proinflammatory cytokine secretion, and hepatic lipid content. ApoE is a class of proteins involved in the metabolism of fats in humans and mice. Its absence predisposes to metabolic syndrome (e.g., Alzheimer's disease, atherosclerosis, and obesity) and might be associated with NAFLD [27]. Therefore, ApoE^−/−^ mice have been extensively employed as models for metabolic syndrome and NAFLD in recent years [28, 29].

It has been reported that consuming DHA and EPA directly from foods and/or dietary supplements is the only practical way to increase the levels of these FAs in the body. The contents of DHA and EPA in the serum and liver tissue of DHA/EPA-treated mice were notably increased in our study. It is also well known that dietary fat, including DHA and EPA, alters the FA composition of various organs [12, 18]. Our results showed that the increased MUFAs and decreased SFAs, n-6 PUFAs, and n-3 PUFAs with an increase of the n-6/n-3 ratio were observed in liver tissue of HFD-fed mice compared to that in the ND-fed mice. This phenomenon is most likely due to the increased activity of Δ-9 desaturase activity [30, 31] and the defective pathway for desaturation and elongation of essential precursors, linoleic acid, and ALA [32]. Our findings are in agreement with the observations of other authors [13, 33]. Interestingly, these changes were either reversed or normalized to the control levels in mice fed the diets supplemented with DHA/EPA (2 : 1, 1 : 1, and 1 : 2). Our study showed that the DHA/EPA 2 : 1 group showed a tendency to raise DHA and n-3 PUFA concentration and lower the n-6/n-3 ratio in the liver. On the other hand, the DHA/EPA 1 : 2 group showed a tendency to raise EPA, n-6 PUFA concentration, and the n-6/n-3 ratio in the liver. The results suggest that DHA/EPA supplementation moderately attenuated the HFD-induced NAFLD, at least partly due to the alteration of FA composition of serum and liver tissue.

The impairment of normal redox homeostasis and the consequent accumulation of oxidized biomolecules have been linked to the onset and/or development of a large variety of diet-induced diseases. An established source of oxidative stress is reactive oxygen species (ROS), which are generated by free FA metabolism and can attack PUFAs and initiate lipid peroxidation within cells. The formation of aldehyde by-products during lipid peroxidation, including MDA, activates the inflammatory response, propagating tissue injury and activating cellular stress signaling pathways. We previously found that the supplementation of various DHA/EPA ratios with an n-6/n-3 ratio of 4 : 1 reversed HFD-induced oxidative stress, as evidenced by the lower content of MDA. These effects are correlated with the induction of serum SOD activity and enhancement in serum levels of GSH and serum total antioxidant capacity, although no significant differences were observed among the DHA/EPA groups (2 : 1, 1 : 1, and 1 : 2) [24]. However, Mendez et al. [21] revealed significant differences in the carbonylation status of albumin in plasma among the DHA/EPA dietary groups, and the EPA : DHA 1 : 1 ratio exhibited the lowest protein oxidation scores. In this study, the general changes in hepatic MDA, SOD, and GSH levels were similar to those observed in our previous report [24]. The difference between the results of our study and those of Mendez et al. may lie in the different FA compositions in the diets. HFD-induced liver oxidative stress is associated with progressively increasing availability and oxidation of FAs in the liver [34] and/or TNF-*α*-induced enhancement in mitochondrial ROS production [35], while the DHA/EPA-reversed liver oxidative stress is possibly related to liver n-6 PUFAs and n-3 PUFA repletion with a decreased n-6/n-3 ratio [36].

Dysfunction of fat storage in adipose tissue may increase adipocyte lipolysis, subsequently causing excessive adipose-derived fatty acid influx into the liver, eventually resulting in hepatic steatosis [37]. By upregulating genes encoding proteins involved in FA oxidation and downregulating genes encoding proteins involved in lipid synthesis, n-3 PUFAs provide their protective effects on NAFLD. SREBP-1c, the key lipogenic transcription factor that is highly expressed in the liver, increases the expression of genes connected with fatty acid and TG synthesis. Our recent study showed that the treatment of C57BL/6J mice with various DHA/EPA ratios repressed SREBP-1c-mediated downregulation of FA synthase, stearoyl desaturase-1, and acetyl-CoA carboxylase with a concomitant reduction in *de novo* lipogenesis and activated PPAR*α*-mediated upregulation of carnitine palmitoyl transferase-1 and acyl-CoA oxidase expression with a parallel enhancement in FA oxidation [17]. As one of the critical adipokines secreted by endocrine organs, adiponectin modulates hepatic lipid homeostasis towards a reduction of lipid content [10]. Activated adiponectin signaling leads to the activation of the AMPK pathway, which modulates hepatic lipid metabolism by simultaneously inhibiting *de novo* lipogenesis and stimulating FA *β*-oxidation [38]. In this study, the reduction of hepatic lipid accumulation in DHA/EPA-treated mice may be attributed to the elevated serum levels of adiponectin. Additionally, mice treated with DHA/EPA showed significant diminution in total liver fat content compared to untreated animals, a finding that may be related to changes in the pattern of lipid metabolism in the liver. To explain the potential mechanism causing the changes, proteins involved in cholesterol efflux (ABCA1 and ABCG1), cholesterol esterification (ACAT1), cholesterol lipolysis (LAL), and cholesterol uptake (CD36, MSR-1, and LOX-1) were examined. This is supported by the higher mRNA expression of the ABCA1, ABCG1, LAL, and ACAT-1 and the lower expression of CD36, MSR-1, and LOX-1. We demonstrated that diets lacking DHA and EPA have no effects on the expression of ABCA1, ABCG1, and LAL, which indicated that DHA and EPA are much more likely to regulate cholesterol homeostasis by increasing cholesterol efflux and lipolysis [24].

In both NAFLD patients and animals subjected to HFD, hepatic proinflammatory status is characterized by Kupffer cell activation, an increased number of hepatic neutrophils, and higher levels of serum transaminases, TNF-*α*, IL-1*β*, and IL-6 [39]. Our recent study showed that serum levels of ALT, AST, TNF-*α*, IL-1*β*, and IL-6 in C57BL/6J mice were all significantly lower in the DHA/EPA groups compared to those in the HFD group [17]. In agreement with these findings, the data presented here show that transaminase activity, TNF-*α*, and IL-1 *β* levels in serum and TNF-*α*, IL-1 *β*, IL-6, MCP-1, VCAM-1, and ICAM-1 mRNA expression in the liver were higher in HFD-fed ApoE^−/−^ mice compared to the controls, a condition that was reverted upon supplementation with various DHA/EPA ratios. Furthermore, mRNA expression of the anti-inflammatory cytokine IL-10 was significantly upregulated by DHA/EPA supplementation. Activating protein-1, including c-Jun and c-Fos, is an important signal transduction pathway component of proinflammatory mediator expression and is independent of NF-*κ*B. We previously found that the consumption of DHA/EPA significantly suppressed the expression of c-Jun and c-Fos protein and their respective genes. Additionally, the critical role of PPAR*α* in preventing fat-induced nonalcoholic steatohepatitis by alleviating liver steatosis, oxidative stress, and inflammation has been proven [40]. The underlying mechanisms by which n-3 PUFAs protected against HFD-induced liver steatosis are probably that n-3 PUFA-activated PPAR*α* interact with proinflammatory factor NF-*κ*B p65 with the formation of inactive PPAR*α*/NF-*κ*B p65 complexes [41] and the suppression of proinflammatory cytokine formation and secretion [7]. Moreover, DHA had a greater suppressive effect than EPA on an alcohol/high-fat diet-induced hepatic inflammation and ROS generation by increasing adiponectin production and secretion [42, 43], which has strong cellular protective properties, acting through the AMPK-activated mechanism [44]. In this study, DHA/EPA supplementation reversed the decrease of hepatic PPAR*α* expression in HFD-fed mice. Although only the DHA/EPA 2 : 1 group had significantly increased PPAR*α* expression, the DHA/EPA 2 : 1 group had the highest serum levels of adiponectin, the lowest hepatic mRNA expression of proinflammatory cytokines, and the highest protein levels of PPAR*α* and AMPK, which may be due to the higher ratio of DHA in this group. These results suggest that the alleviation of inflammatory responses in DHA/EPA-treated mice may correlate with an increase in serum levels of adiponectin and hepatic protein levels of PPAR*α* and AMPK.

## 5. Conclusion

In addition to reducing oxidative stress, decreasing proinflammatory cytokine secretion, and improving hepatic lipid metabolism, a DHA/EPA-enriched diet with an n-6/n-3 ratio of 4 : 1 may reverse HFD-induced NALFD to some extent by increasing n-6 and n-3 PUFAs and decreasing the amount of MUFAs and the n-6/n-3 ratio. Although no significant difference was found in the expression of inflammation- and hepatic lipid metabolism-related genes in the three DHA/EPA groups, the DHA/EPA 2 : 1 group showed the highest DHA and n-3 PUFA concentration and the DHA/EPA 1 : 2 group showed the highest EPA, n-6 PUFA concentration, and n-6/n-3 ratio.

## Figures and Tables

**Table 1:** Fatty acid composition of oils and feed supplemented to mice.

Fatty acid	mg per 100 mg total fatty acid									
	Sunflower seed oil	Perilla oil	Fish oil	Algal oil	Basic diet	HFD I	HFD II	DHA/EPA = 2 : 1	DHA/EPA = 1 : 1	DHA/EPA = 1 : 2
C14:0	0.1	0	0.3	8.7	0	0	0	1.4	0.8	0.1
C14:1	0	0	0.2	0.3	0	0	0	0.1	0.1	0.1
C15:0	0	0	0.5	0.2	0	0	0	0.1	0.1	0.1
C16:0	6.5	0.4	10.1	15.8	27.0	42.0	43.7	8.6	8.1	8.0
C16:1	0.1	0.1	4.7	3.5	0	1.8	1.7	1.1	1.2	1.5
C17:0	0	0	0.1	0.3	0	0	0	0.1	0.1	0
C17:1	0	0	0.1	0	0	0	0	0	0	0
C18:0	5.2	0	2.8	0	4.7	0.2	0.3	4.4	4.6	4.9
C18:1	24.9	10.1	7.9	13.5	21.2	16.5	15.6	22.2	22.4	22.2
C18:2	60.7	15.4	1.9	1.6	36.9	21.5	21.7	48.4	49.3	49.9
C18:3	0	73.3	2.0	0	0	16.0	15.3	0.2	0.4	0.2
C20:1	0	0	0.1	0.3	0	0.13	0.12	0.1	0.1	0
C20:2	0	0	0.2	0	0	0	0	0	0	0.1
C20:4	0	0	1.5	0.5	0.5	0.19	0.20	0.2	0.3	0.4
C20:5	0.2	0	35.1	0.0	0	0	0	4.3	6.2	8.1
C22:1	0	0	0.3	3.5	0	0	0	0.5	0.3	0.1
C22:5	0	0	2.4	0.0	0	0	0	0.3	0.4	0.6
C22:6	0	0	18.2	40.1	0	0	0	8.0	6.0	4.3
∑SATs	11.8	0.4	13.8	25	31.7	42.2	44	14.6	13.7	13.1
∑MUFAs	25	10.2	13.3	21.1	21.2	18.43	17.42	24	24.1	23.9
∑PUFAs	60.9	88.7	61.3	42.2	37.4	37.69	37.2	61.4	62.6	63.6
∑n-6	60.7	15.4	3.4	2.1	36.9	21.5	21.7	48.6	49.6	50.3
∑n-3	0.2	73.3	57.7	40.1	0	16.0	15.3	12.8	13.0	13.2
n-6/n-3						1.3	1.4	3.9	3.8	3.8
EPA/DHA	0	0	1.9	0	0	0	0	1.9	1.0	0.5

**Table 2:** Quantitative PCR primer sequences.

Gene	Forward primer 5′–3′	Reverse primer 5′–3′
IL-6	TCCAGTTGCCTTCTTGGGAC	AGTCTCCTCTCCGGACTTGT
IL-10	GCTGCCTGCTCTTACTGACT	CTGGGAAGTGGGTGCAGTTA
IL-1*β*	TGCCACCTTTTGACAGTGATG	TGATGTGCTGCTGCGAGATT
TNF-*α*	ATGGCCTCCCTCTCATCAGT	TTTGCTACGACGTGGGCTAC
MCP-1	TATTGGCTGGACCAGATGCG	CCGGACGTGAATCTTCTGCT
VCAM-1	CTGGGAAGCTGGAACGAAGT	GCCAAACACTTGACCGTGAC
ICAM-1	TATGGCAACGACTCCTTCT	CATTCAGCGTCACCTTGG
CD36	CGGGCCACGTAGAAAACACT	CAGCCAGGACTGCACCAATA
MSR-1	GACTTCGTCATCCTGCTCAAT	GCTGTCGTTCTTCTCATCCTC
LOX-1	TCACCTGCTCCCTGTCCTT	GGTTCTTTGCCTCAATGCC
ABCA-1	CGACCATGAAAGTGACACGC	AGCACATAGGTCAGCTCGTG
ABCG-1	AGAGCTGTGTGCTGTCAGTC	AGCAGGTCTCAGGGTCTAGG
LAL	CCCACCAAGTAGGTGTAGGC	GAGTTGCATCGGGAGTGGTC
ACAT-1	CCAATGCCAGCACACTGAAC	TCTACGGCAGCATCAGCAAA
*β*-Actin	TTCGTTGCCGGTCCACACCC	GCTTTGCACATGCCGGAGCC

**Table 3:** Effects of DHA/EPA supplementation on body and liver weights in each group.

	Initial weight (g)	Final weight (g)	Weight gain (g)	Liver weight (g)	Liver ratio to weight (%)
ND	20.6 ± 1.2	25.4 ± 2.8	4.8 ± 3.0	1.03 ± 0.23	4.2 ± 0.4
HFD	20.5 ± 1.1	27.0 ± 2.5	6.5 ± 2.7	1.15 ± 0.17	4.5 ± 0.4
DHA/EPA = 2 : 1	20.8 ± 1.0	26.8 ± 2.5	6.0 ± 2.5	1.10 ± 0.21	4.3 ± 0.4
DHA/EPA = 1 : 1	20.7 ± 1.2	25.8 ± 2.6	4.9 ± 2.6	1.04 ± 0.20	4.2 ± 0.4
DHA/EPA = 1 : 2	20.3 ± 1.5	25.6 ± 2.4	5.3 ± 2.5	1.06 ± 0.15	4.3 ± 0.3

Data are given as the mean ± SEM, *n* = 10.

**Table 4:** General and biochemical parameters in serum and liver tissues.

	ND	HFD	DHA/EPA = 2 : 1	DHA/EPA = 1 : 1	DHA/EPA = 1 : 2
Serum parameters					
TC (mM)	9.50 ± 0.46	19.38 ± 0.66^a^	5.43 ± 0.52^a,b^	7.82 ± 0.84^b,c^	10.29 ± 0.31^b,c,d^
TG (mM)	1.19 ± 0.05	2.38 ± 0.24^a^	0.58 ± 0.05^a,b^	0.75 ± 0.08^a,b^	1.30 ± 0.07^b,c,d^
LDL (mM)	3.51 ± 0.19	7.03 ± 0.46^a^	2.55 ± 0.43^b^	4.17 ± 0.40^b,c^	4.34 ± 0.17^a,b,c^
HDL (mM)	0.30 ± 0.03	0.13 ± 0.03^a^	0.26 ± 0.03^a,b^	0.35 ± 0.05^b^	0.21 ± 0.01^a,b,d^
Adiponectin (pg/mg)	159.76 ± 23.19	81.64 ± 8.36^a^	196.77 ± 18.68^b^	114.91 ± 14.16^c^	103.97 ± 7.43^b,c^
OX-LDL (*μ*g/L)	223.46 ± 25.32	269.00 ± 14.73	171.58 ± 8.58^b^	165.90 ± 8.29^b^	165.89 ± 10.76^a,b^
AST (U/L)	143.79 ± 21.97	487.5 ± 95.19^a^	63.62 ± 7.36^a,b^	110.23 ± 13.31^b,c^	138.32 ± 28.99^b,c^
ALT (U/L)	72.92 ± 9.06	210.82 ± 23.72^a^	40.69 ± 4.88^a,b^	51.67 ± 3.05^a,b^	93.06 ± 13.03^b,c,d^
AKP (U/L)	48.94 ± 5.18	90.66 ± 7.22^a^	42.08 ± 3.50^b^	58.93 ± 5.92^b,c^	70.34 ± 4.55^a,b,c^
Liver parameters					
TC (mM/g protein)	74.94 ± 3.62	144.57 ± 4.72^a^	73.81 ± 4.22^b^	83.72 ± 3.30^b,c^	77.59 ± 6.03^b^
TG (mM/g protein)	204.01 ± 25.74	231.19 ± 14.54	125.21 ± 14.26^a,b^	114.81 ± 7.32^a,b^	160.34 ± 15.76
MDA (*μ*M/g protein)	1.75 ± 0.17	2.31 ± 0.18^a^	1.81 ± 0.14^b^	1.73 ± 0.07^b^	1.90 ± 0.08
SOD (U/mg protein)	6.2 ± 0.32	5.79 ± 0.16	6.9 ± 0.14^b^	6.85 ± 0.03^a,b^	6.86 ± 0.26^b^
GSH (*μ*M/g protein)	19.46 ± 2.37	10.8 ± 1.57^a^	21.9 ± 2.59^b^	23.81 ± 1.86^b^	24.33 ± 2.69^b^

Data are given as mean ± SEM, *n* = 8. ^a^*P* < 0.05 versus the ND group; ^b^*P* < 0.05 versus the HFD group; ^c^*P* < 0.05 versus the DHA/EPA = 2 : 1 group; ^d^*P* < 0.05 versus the DHA/EPA = 1 : 1 group.

**Table 5:** Fatty acid composition (%) of the serum of mice during the experimental period.

Serum fatty acid	ND (*n* = 5)	HFD (*n* = 5)	DHA/EPA = 2 : 1 (*n* = 4)	DHA/EPA = 1 : 1 (*n* = 5)	DHA/EPA = 1 : 2 (*n* = 5)
C16:0	22.881 ± 0.863	23.293 ± 0.271	22.759 ± 0.762	22.068 ± 0.763	20.838 ± 1.006
C16:1	1.069 ± 0.117	1.473 ± 0.145^a^	0.773 ± 0.287^b^	0.65 ± 0.168^b^	0.666 ± 0.174^b^
C18:0	8.151 ± 0.345	11.729 ± 0.440^a^	9.163 ± 0.510^b^	9.071 ± 0.336^b^	8.871 ± 0.576^b^
C18:1	16.136 ± 0.603	26.315 ± 0.857^a^	17.374 ± 1.279^b^	16.886 ± 1.21^b^	16.174 ± 1.967^b^
C18:2	30.819 ± 1.416	20.912 ± 0.605^a^	30.538 ± 1.028^b^	30.515 ± 1.039^b^	28.062 ± 1.046^a,b^
C18:3	0.00 ± 0.00	0.00 ± 0.00	0.04 ± 0.08	0.00 ± 0.00	0.00 ± 0.00
C19:0	0.836 ± 0.040	0.538 ± 0.137	0.500 ± 0.168	0.266 ± 0.1646^a^	0.260 ± 0.162^a^
C20:0	0.112 ± 0.112	0.082 ± 0.0.082	0.133 ± 0.133	0.098 ± 0.098	0.00 ± 0.00
C20:1	0.106 ± 0.106	0.228 ± 0.140	0.748 ± 0.329	0.416 ± 0.289	0.424 ± 0.309
C20:4	4.265 ± 0.272	7.177 ± 0.458^a^	3.67 ± 0.132^b^	3.946 ± 0.126^b^	4.242 ± 0.184^b^
C22:0	2.01 ± 0.059	0.660 ± 0.093	4.018 ± 1.264^a,b^	4.383 ± 0.518^a,b^	5.472 ± 0.775^a,b^
C20:5	0.752 ± 0.313	0.00 ± 0.00^a^	0.275 ± 0.166	0.102 ± 0.102^a^	0.130 ± 0.130^a^
C22:6	7.028 ± 0.305	3.555 ± 0.221^a^	8.389 ± 0.371^a,b^	8.347 ± 0.265^a,b^	7.358 ± 0.429^b,c,d^
∑SFAs	33.987 ± 1.153	36.305 ± 0.321	36.575 ± 0.697	35.885 ± 0.986	35.439 ± 0.969
∑MUFAs	17.314 ± 0.598	28.018 ± 0.805^a^	18.895 ± 1.131^b^	17.952 ± 1.090^b^	17.266 ± 1.845^b^
∑PUFAs	42.866 ± 1.969	31.648 ± 0.510^a^	42.878 ± 1.189^b^	42.908 ± 0.721^b^	39.794 ± 1.124^b^
∑n-6	35.086 ± 1.659	28.092 ± 0.339^a^	34.213 ± 1.021^b^	34.460 ± 0.927^b^	32.306 ± 1.134^b^
∑n-3	7.780 ± 0.393	3.556 ± 0.222^a^	8.665 ± 0.493^b^	8.448 ± 0.253^b^	7.488 ± 0.388^b,c^
n-6/n-3	4.522 ± 0.162	8.016 ± 0.469^a^	3.983 ± 0.241^b^	4.105 ± 0.224^b^	4.372 ± 0.320^b^

Data are given as the mean ± SEM. ^a^*P* < 0.05 versus the ND group; ^b^*P* < 0.05 versus the HFD group; ^c^*P* < 0.05 versus the DHA/EPA = 2 : 1 group; ^d^*P* < 0.05 versus the DHA/EPA = 1 : 1 group.

**Table 6:** Fatty acid composition (%) of the liver of mice during the experimental period.

Hepatic fatty acid	ND (*n* = 5)	HFD (*n* = 4)	DHA/EPA = 2 : 1 (*n* = 5)	DHA/EPA = 1 : 1 (*n* = 4)	DHA/EPA = 1 : 2 (*n* = 3)
C16:0	26. 228 ± 0.799	20.211 ± 0.217^a^	22.596 ± 0.689^a^	21.261 ± 1.272^a^	20.590 ± 0.116^a^
C16:1	0.00 ± 0.000	1.640 ± 0.247^a^	0.48 ± 0.045^a,b^	0.489 ± 0.104^a,b^	0.577 ± 0.044^a,b^
C18:0	10.905 ± 0.797	8.772 ± 0.399	11.234 ± 0.922	10.545 ± 1.303	7.260 ± 0.297^a,c,d^
C18:1	13.451 ± 0.814	30.939 ± 0.911^a^	15.386 ± 1.691^b^	17.971 ± 3.113^b^	20.368 ± 1.028^a,b^
C18:2	25.962 ± 0.722	19.456 ± 0.555^a^	24.647 ± 0.708^b^	23.941 ± 0.966^b^	26.889 ± 0.404^b,d^
C18:3	0.452 ± 0.029	0.546 ± 0.059	0.099 ± 0.011^a,b^	0.231 ± 0.049^a,b,c^	0.133 ± 0.009^a,b^
C19:0	0.397 ± 0.049	0.293 ± 0.093	0.284 ± 0.057	0.188 ± 0.069^a^	0.334 ± 0.024
C20:0	0.358 ± 0.019	0.617 ± 0.208	0.333 ± 0.026^b^	0.431 ± 0.043	0.391 ± 0.032
C20:1	0.710 ± 0.074	0.689 ± 0.021	0.879 ± 0.099	0.894 ± 0.118	0.567 ± 0.041^c,d^
C20:4	6.371 ± 0.435	7.525 ± 0.381	5.332 ± 0.388^b^	5.489 ± 0.738^b^	4.932 ± 0.335^b^
C22:0	0.880 ± 0.041	0.156 ± 0.012^a^	1.506 ± 0.046^a,b^	1.98 ± 0.081^a,b,c^	1.800 ± 0.138^a,b,c^
C20:5	0.804 ± 0.064	0.451 ± 0.076^a^	1.086 ± 0.056^a,b^	1.315 ± 0.074^a,b,c^	1.527 ± 0.096^a,b,c^
C22:6	10.425 ± 0.223	4.289 ± 0.281^a^	12.895 ± 0.530^a,b^	11.761 ± 1.000^b^	10.301 ± 0.483^b,c^
∑SFAs	38.770 ± 1.345	30.053 ± 0.432^a^	35.948 ± 1.489^b^	34.403 ± 2.263^a^	30.377 ± 0.126^a,c^
∑MUFAs	14.166 ± 0.782	33.269 ± 1.066^a^	16.746 ± 1.644^b^	19.358 ± 3.044^a,b^	21.517 ± 1.032^a,b^
∑PUFAs	44.016 ± 0.467	32.268 ± 0.757^a^	44.064 ± 0.578^b^	42.738 ± 0.826^b^	43.787 ± 0.492^b^
∑n-6	32.334 ± 0.643	26.983 ± 0.484^a^	29.982 ± 0.383^a,b^	29.43 ± 0.249^a,b^	31.823 ± 0.143^b,c,d^
∑n-3	11.682 ± 0.255	5.285 ± 0.293^a^	14.082 ± 0.519^a,b^	13.308 ± 1.069^b^	11.963 ± 0.387^b,c^
n-6/n-3	2.778 ± 0.116	5.138 ± 0.202^a^	2.141 ± 0.081^a,b^	2.266 ± 0.228^a,b^	2.665 ± 0.082^b,c^

Data are given as mean ± SEM. ^a^*P* < 0.05 versus the ND group; ^b^*P* < 0.05 versus the HFD group; ^c^*P* < 0.05 versus the DHA/EPA = 2 : 1 group; ^d^*P* < 0.05 versus the DHA/EPA = 1 : 1 group.

## Data Availability

The data used to support the findings of this study are available from the corresponding author upon request.
